# Podcasts and YouTube Videos: Innovative Tools for Disseminating Mental Health and Dementia Education to the Public

**DOI:** 10.1093/geroni/igab046.1942

**Published:** 2021-12-17

**Authors:** Regina Koepp, Natali Edmonds

## Abstract

This symposium will discuss two examples of innovative public education tools used to disseminate evidence-based information to the general public about mental health and aging and Alzheimer's Disease and related dementias. The first is the Psychology of Aging Podcast hosted by Regina Koepp, Clinical Geropsychologist. Since it’s launch in April 2020, there have been 50 weekly episodes and more than total 25,000 downloads. The goal of the Psychology of Aging podcast is to facilitate access to information and education about mental health and brain health among older adults with the hope of de-stigmatizing mental health care for older adults, reducing ageism, and promoting access to mental health and dementia care for older adults and their families. The second is Dementia Careblazers, created and hosted by Dr. Natali Edmonds, board certified Geropsychologist. The goal of Dementia Careblazers videos is to offer easy to access information to family members who care for someone with dementia. In her weekly videos, Dr. Edmonds provides actionable, evidence-based information and resources focused on dementia caregiving in brief videos. Since it’s launch on YouTube November 2016, Dementia Careblazers, has 65,000 subscribers, has posted 231 videos, and has had more than 4 million views nationally and internationally. Drs. Koepp and Edmonds will discuss the role podcasts and YouTube videos play in public education and share tips for professionals wanting to start an evidence-based program of their own. This virtual modality may be of increased interest considering recent health risks through face to face interactions and advancements in technology.

